# Heart Failure in Atrial Fibrillation Subtypes in Women and Men in the Tromsø Study

**DOI:** 10.1016/j.jacadv.2024.101556

**Published:** 2025-01-10

**Authors:** Hilde Espnes, Tom Wilsgaard, Jocasta Ball, Maja-Lisa Løchen, Inger Njølstad, Renate B. Schnabel, Eva Gerdts, Ekaterina Sharashova

**Affiliations:** a Department of Community Medicine, UiT The Arctic University of Norway, Tromsø, Norway; b School of Public Health and Preventive Medicine, Monash University, Melbourne, Australia; c Department of Clinical Medicine, UiT The Arctic University of Norway, Tromsø, Norway; d Department of Cardiology, University Heart and Vascular Centre Hamburg-Eppendorf, Hamburg, Germany; e German Centre for Cardiovascular Research (DZHK), Partner Site Hamburg/Kiel/Luebeck, Berlin, Germany; f Department of Clinical Science, University of Bergen, Bergen, Norway

**Keywords:** atrial fibrillation, cohort study, heart failure, sex differences

## Abstract

**Background:**

Atrial fibrillation (AF) and heart failure (HF) often coexist and impact morbidity and mortality. There is limited knowledge on the association of AF subtypes with HF according to sex.

**Objectives:**

The purpose of this study was to explore sex-specific associations between AF subtypes and subsequent HF, identifying HF risk factors in participants with AF, and exploring the combined impact on mortality.

**Methods:**

14,790 women and 13,181 men from the Tromsø Study were enrolled between 1994 and 2008 and followed for incident AF and HF through 2016. Cox regression was conducted to provide HRs and 95% CIs.

**Results:**

Those with AF had higher risk of subsequent HF in both sexes compared to those without AF. Women with permanent AF had higher relative risk of HF than men (HR: 10.52; 95% CI: 8.72-12.70, and HR: 7.65; 95% CI: 6.40-9.15, respectively). Risk factors for HF in participants with AF included smoking in all, higher diastolic blood pressure and hypertension in women, underweight, obesity, and low alcohol consumption in men. All-cause mortality was higher in women with both subtypes (paroxysmal/persistent: HR: 2.10; 95% CI: 1.78-2.48, permanent: HR: 1.40, 95% CI: 1.14-1.72) and in men with paroxysmal/persistent AF (HR: 1.66; 95% CI: 1.40-1.96). Subsequent HF increased risk of mortality in both sexes.

**Conclusions:**

All AF subtypes were associated with increased risk of HF. Smoking was a shared risk factor, while diastolic blood pressure and hypertension were specific to women, and underweight, obesity, and low alcohol intake were specific to men. Subsequent HF increased mortality risk in all.

Atrial fibrillation (AF) and heart failure (HF) are common diseases that impact morbidity, mortality, and quality of life.^1^^,^^2^ Both conditions primarily affect the elderly, and their prevalence is on the rise due to aging of the population and an increase in metabolic risk factors like hypertension, obesity, and diabetes.^3^^,^^4^

There is a bidirectional relationship between AF and HF, with over one-third of individuals with AF developing HF, and about half of those with HF developing AF.^5^ AF and HF share common risk factors and underlying conditions, exacerbating each other, and worsening patient prognosis.^6^^,^^7^ HF accounts for almost a third of all deaths in the first year after AF onset,^8^ which emphasizes the importance of preventing HF in patients with AF.

Sex differences in the development and prognosis of both AF and HF have been reported.^1^^,^^9^, ^10^, ^11^, ^12^ Women with AF face a higher risk of HF and have a poorer prognosis when both conditions coexist.^13^ Additionally, nonparoxysmal AF has been associated with an increased risk of adverse outcomes compared to paroxysmal AF when AF precedes HF.^14^^,^^15^ However, there is a need for better understanding of how modifiable risk factors contribute to the risk of HF in women and men with AF and whether the subsequent risk of mortality varies across subtypes of AF. Our study aims to address these knowledge gaps by exploring sex-specific associations between AF subtypes and subsequent HF, identifying underlying risk factors for HF in participants with AF, and exploring the combined impact on mortality in both sexes.

## Materials and methods

### Study design and participants

Participants were recruited from the fourth (Tromsø4, 1994-1995), fifth (Tromsø5, 2001), and sixth (Tromsø6, 2007-2008) surveys of the Tromsø Study, a longitudinal cohort study in the municipality of Tromsø, Northern Norway.^16^ In these surveys, the whole population (Tromsø4) or parts of the population (Tromsø5 and Tromsø6) aged ≥25 years were invited to participate. The number of attendees was 27,158 (72%) in Tromsø4, 8,130 (79%) in Tromsø5, and 12,984 (66%) in Tromsø6. A total of 30,288 inhabitants aged 25 to 97 years attended ≥1 survey. We excluded individuals who emigrated before the examination date (n = 21), had a previous history of HF (n = 134) or AF (n = 219), had insufficient information for AF validation (n = 1,919), or were diagnosed with AF and HF on the same day (n = 24) (Supplemental Figure 1). The total study population consisted of 14,790 women and 13,181 men.

### Data collection

Information was collected through questionnaires, physical examinations, and blood samples.^17^ Resting heart rate (beats/min), systolic blood pressure (BP) (mm Hg), and diastolic BP (mm Hg) were measured by trained personnel using an automated Dinamap device.^17^ The mean of the last two of three measurements was used in the current analyses. Hypertension was defined as systolic BP ≥140 mm Hg, or diastolic BP ≥90 mm Hg, or current use of antihypertensive medication. Weight and height were measured and used to calculate body mass index (BMI) (kg/m^2^). Nonfasting serum total cholesterol (mmol/L), high-density lipoprotein cholesterol (mmol/L), and triglycerides (mmol/L) were analyzed by the Department of Laboratory Medicine, University Hospital of North Norway.

Information on current use of antihypertensive medications (yes/no), current daily smoking (yes/no), alcohol consumption, physical activity, and history of myocardial infarction (yes/no), angina pectoris (yes/no), stroke (yes/no), and diabetes mellitus (yes/no) was obtained from questionnaires. Alcohol consumption in Tromsø4 and Tromsø5 was assessed using questions regarding how many glasses of beer/wine/spirits participants drank in a fortnight. In Tromsø6, participants reported the frequency of drinking and number of units (a beer, a glass of wine, or a drink) consumed per occasion. Consumption was categorized into 0 units per week, <1 unit per week, 1 to 2 units per week, 3 to 4 units per week, and ≥5 units per week.

Physical activity was categorized into sedentary, moderate, and highly active, using participant-reported levels of exercise and physical exertion in leisure time over the last 12 months. For participants in Tromsø4 and those aged ≥70 years in Tromsø5, 2 questions on the number of hours per week of light (not sweating or out of breath) and hard (sweating/out of breath) activity were recoded to correspond to the 3 levels.^18^

### Follow-up and detection of incident AF and HF

Follow-up of participants began on the date of the first examination and ended on the date of first documented HF, emigration, death (identified through the Population Register of Norway), or the end of the follow-up period (December 31, 2016), whichever came first. Start of follow-up was changed to the date of AF in analyses restricted to the subgroup of participants with this diagnosis. In mortality analyses, those who developed HF without a prior AF diagnosis were censored at the date of HF, while for the other participants, the end of follow-up was set to the first documentation of either emigration, death, or the end of the follow-up period.

Using the unique Norwegian national identification number, incident AF and HF were detected through linkage to the diagnosis registry at the University Hospital of North Norway. This registry includes diagnoses from both outpatient and inpatient clinics. By using the International Classification of Diseases-9th Revision (ICD-9) codes 427.0 to 427.99 and -10th Revision (ICD-10) codes I47 and I48, participants with a diagnosis of AF were identified. Incident HF was identified using the ICD-9 code 428 and ICD-10 code I50. In addition, for participants with a diagnosis of a cardiovascular event but no recorded diagnosis of arrhythmia, hospital records were searched for AF. Diagnosis of AF was confirmed if documented on an electrocardiogram and validated by an independent endpoint committee following a detailed protocol.^19^ The latest AF subtype recorded was used and classified according to the 2016 European Society of Cardiology guidelines for the management of AF^20^: paroxysmal (self-terminating and lasting ≤7 days); persistent (lasting >7 days, including episodes requiring intervention to terminate); and permanent (sustained AF). Data on long-term monitoring of cardiac rhythm were not available and, therefore, paroxysmal and persistent AF were combined. Transient AF occurring within 28 days after myocardial infarction/acute cardiac event or cardiac surgery, and AF occurring during the last 7 days of life, were not classified as AF cases.

### Statistical analyses

Sex-specific characteristics of the study population at first attendance are presented as mean ± SD for continuous variables and as numbers (percentages) for categorical variables. Means and proportions in AF subgroups (no AF, paroxysmal/persistent AF, and permanent AF) were adjusted for age using linear regression for continuous variables and logistic regression for categorical variables and estimated for the overall average age of 45 years. Cox proportional hazards regression models were used to estimate sex-specific HRs and 95% CIs for the association between AF subtypes and the risk of incident HF, to explore modifiable risk factors for HF in participants with and without AF, as well as the risk of mortality when AF preceded HF. All models were adjusted for systolic BP, BMI, total cholesterol, smoking status, physical activity level, alcohol consumption, history of myocardial infarction, stroke, and diabetes mellitus, and for age using age as the time scale. When we investigated systolic BP, diastolic BP, and hypertension as risk factors for HF, only one of these measurements was included in the models at a time. In the analyses investigating risk factors for HF in participants with AF, values of the covariates at the closest survey conducted prior to the AF diagnosis were used. To account for competing risk of death, the Fine-Gray subdistribution hazard model was conducted for both the association between AF subtypes and HF, and for exploring risk factors of HF in AF participants. To assess the possibility of the multiple comparisons problem in the risk factors analyses, False Discovery Rate adjusted *P* values (q-values) were calculated using the Benjamini-Hochberg procedure.

Incidence rates and mortality rates with 95% CIs were estimated per 1,000 person-years and adjusted for the overall average age of 45 years using Poisson regression. To prevent immortal time bias, we modeled AF or HF as time dependent covariates. The data set was structured with one extra record for participants who developed AF (or HF in the mortality analyses) during follow-up, ie, if a participant developed AF, they contributed one record before AF onset and another after AF onset. The same individual contributed with only one main outcome in each analysis. To explore the risk of all-cause and cardiovascular mortality, participants were divided into 5 groups based on which AF subtype they developed and if HF succeeded AF. Participants without AF and HF were used as the reference. Tests for interaction with sex were performed by including cross-product terms in all models. To further assess the temporal relationship between AF subtypes and incident HF and all-cause mortality in AF participants with and without subsequent HF, we included graphical presentations of cumulative incidence of HF and cumulative probability of mortality estimated from Cox regression with time under study as the time scale. Models were adjusted for age at start of follow-up and the covariates above.

The proportional hazard assumption was assessed with graphical inspection of log minus log survival curves between quartiles of continuous variables or between categories of nominal variables. All analyses were performed using SAS, 9.4 (SAS Institute) and a 2-sided *P* value <0.05 was considered statistically significant.

## Results

### Baseline characteristics

Over a median follow-up of 21.6 years (25th, 75th percentile: 10.2, 22.0 years), 848 women (467 paroxysmal/persistent AF and 381 permanent AF) and 1,020 men (582 paroxysmal/persistent AF and 438 permanent AF) developed AF (Table 1). Women who developed permanent AF were older and had higher age-adjusted mean systolic BP and BMI, compared to women who developed paroxysmal/persistent AF. Prevalence of hypertension and usage of antihypertensive medications were higher among women who developed permanent AF. Men who developed permanent AF were older compared to those who developed paroxysmal/persistent AF. Unlike women, men who developed permanent AF had lower mean systolic BP and diastolic BP than men who developed paroxysmal/persistent AF, and the proportions with hypertension and use of antihypertensive medications were nearly identical between the AF subtypes.

**Table 1:** Baseline Characteristics of Study Participants According to Sex and Atrial Fibrillation Subtype: The Tromsø Study, 1994 to 2016

	Women (n = 14,790)				Men (n = 13,181)			
	No AF	Parox/Pers	Perm		No AF	Parox/Pers	Perm	
	(n = 13,942, 94.3)	(n = 467, 3.1)	(n = 381, 2.6)	*P* Value	(n = 12,161, 92.3)	(n = 582, 4.4)	(n = 438, 3.3)	*P* Value
Age, y	44.5 (13.8)	61.9 (12.1)	67.1 (9.2)	<0.001	44.0 (12.8)	56.0 (12.5)	60.1 (10.9)	<0.001
Systolic blood pressure, mm Hg	129.2 (20.3)	136.1 (24.9)	138.2 (24.4)	<0.001	135.7 (16.1)	140.2 (20.8)	139.1 (21.1)	<0.001
Diastolic blood pressure, mm Hg	75.2 (11.7)	78.3 (14.0)	78.4 (13.4)	<0.001	79.3 (11.1)	81.2 (12.2)	80.9 (13.0)	<0.001
Hypertension^a^	3,433 (19.3)	307 (28.6)	299 (31.5)	<0.001	4,409 (36.5)	340 (44.7)	277 (44.8)	<0.001
Antihypertensive medication use	649 (2.8)	79 (4.1)	102 (5.7)	<0.001	585 (3.3)	82 (5.0)	72 (4.8)	<0.001
Body mass index, kg/m^2^	24.8 (4.2)	25.4 (4.5)	26.6 (5.2)	<0.001	25.7 (3.4)	26.2 (3.7)	26.9 (3.6)	<0.001
Resting heart rate, beats/min	74.1 (11.9)	74.6 (13.4)	73.3 (13.1)	0.263	70.3 (12.0)	70.4 (12.9)	69.3 (12.9)	0.477
Total cholesterol, mmol/L	5.91 (1.34)	6.03 (1.33)	5.86 (1.22)	0.060	5.96 (1.21)	5.97 (1.13)	5.94 (1.13)	0.912
HDL cholesterol, mmol/L	1.64 (0.40)	1.62 (0.41)	1.60 (0.48)	0.265	1.34 (0.35)	1.32 (0.35)	1.33 (0.37)	0.358
Triglycerides, mmol/L	1.31 (0.81)	1.40 (1.00)	1.44 (1.09)	0.001	1.77 (1.17)	1.77 (1.00)	1.83 (1.18)	0.603
Current daily smoking	5,066 (36.0)	150 (38.7)	72 (25.3)	<0.001	4,493 (36.8)	166 (30.0)	127 (31.0)	<0.001
Alcohol consumption, U/week								
0 U/week	5,043 (36.6)	263 (41.0)	244 (44.1)	0.006	2,452 (20.2)	171 (22.4)	133 (21.4)	0.365
<1 U/week	1,407 (10.2)	33 (8.1)	26 (8.2)	0.787	803 (6.7)	40 (6.0)	33 (6.3)	0.834
1-2 U/week	4,551 (32.3)	97 (28.6)	76 (30.6)	0.195	3,477 (29.0)	150 (26.3)	118 (27.9)	0.601
3-4 U/week	1,780 (12.5)	42 (12.4)	18 (7.4)	0.260	2,526 (20.6)	82 (16.2)	76 (21.2)	0.096
5 or more U/week	948 (6.8)	26 (6.7)	13 (4.3)	0.258	2,728 (22.2)	139 (27.5)	74 (21.1)	0.016
Leisure time physical activity								
Sedentary	4,879 (35.5)	233 (38.2)	207 (39.0)	0.212	3,379 (28.2)	173 (24.9)	137 (24.9)	0.071
Moderate active	7,793 (56.5)	208 (53.2)	157 (52.3)	0.144	6,561 (54.4)	329 (57.9)	247 (58.6)	0.080
Highly active	1,073 (7.1)	21 (7.2)	13 (6.6)	0.957	2,096 (16.5)	77 (16.5)	49 (15.6)	0.907
History of myocardial infarction	118 (0.3)	16 (0.4)	12 (0.3)	0.554	304 (1.3)	33 (1.2)	35 (1.4)	0.798
History of angina pectoris	284 (0.6)	46 (1.0)	41 (0.8)	0.047	331 (1.3)	63 (2.4)	52 (2.2)	<0.001
History of stroke	119 (0.5)	14 (0.7)	9 (0.5)	0.466	137 (0.7)	8 (0.4)	22 (1.3)	0.011
History of diabetes mellitus	174 (0.9)	27 (1.9)	14 (1.0)	0.004	164 (1.0)	19 (1.1)	11 (0.7)	0.493

AF = atrial fibrillation; HDL = high-density lipoprotein; parox = paroxysmal AF; pers = persistent AF; perm = permanent AF.

Values are mean ± SD or n (%); the means (except age means) and percentages are adjusted for age using linear or logistic regression models, respectively, and estimated for a mean age of 45 years. Due to missing, the number of observations may be marginally different for each variable.

a Hypertension was defined as systolic blood pressure ≥140 mm Hg, or diastolic blood pressure ≥90 mm Hg, or current use of antihypertensive medications.

### AF subtypes and incident HF

Incident HF occurred in 753 women and 914 men, and the incidence rate per 1,000 person-years ranged from 0.9 (95% CI: 0.8-1.0) and 1.9 (95% CI: 1.7-2.2) in those without AF to 9.4 (95% CI: 7.6-11.6) and 16.7 (95% CI: 14.0-20.0) in those with permanent AF for women and men, respectively (Table 2). Compared to participants without AF, those with paroxysmal/persistent AF had a higher subsequent HF risk in both sexes. The risk was even higher in those with permanent AF, where women had a significantly higher HR than men. Cumulative incidence of HF by AF subtype over the observational period reflects the same findings (Supplemental Figure 2). These results also remained consistent when accounting for the competing risk of death (Supplemental Table 1).

**Table 2:** HRs of Incident Heart Failure According to Atrial Fibrillation Subtype by Sex: The Tromsø Study, 1994 to 2016

	Women (n = 14,790)		*P* Value	Men (n = 13,181)			*P* Value^b^
	IR (95% CI)^a^	HR (95% CI)		IR (95% CI)^a^	HR (95% CI)	*P* Value	
Participants without AF	0.9 (0.8-1.0)	1.00 (Reference)		1.9 (1.7-2.2)	1.00 (Reference)		
Paroxysmal/persistent AF	6.4 (5.0-8.0)	5.48 (4.39-6.84)	<0.001	12.8 (10.5-15.6)	5.82 (4.78-7.09)	<0.001	0.829
Permanent AF	9.4 (7.6-11.6)	10.52 (8.72-12.70)	0.001	16.7 (14.0-20.0)	7.65 (6.40-9.15)	<0.001	<0.001

IR = incidence rate; other abbreviation as in Table 1.

HRs are adjusted for systolic blood pressure, body mass index, serum total cholesterol, current smoking, alcohol consumption, physical activity, and history of myocardial infarction, stroke, and diabetes mellitus, as well as age by using age as the time scale in the Cox regression models.

a IR per 1,000 person-years, adjusted for a mean age of 45 years and calculating 95% CIs by using Poisson regression.

b *P* value for the difference between sexes calculated by including cross-product terms in the models.

### Modifiable risk factors for incident HF

**Figure 1:**
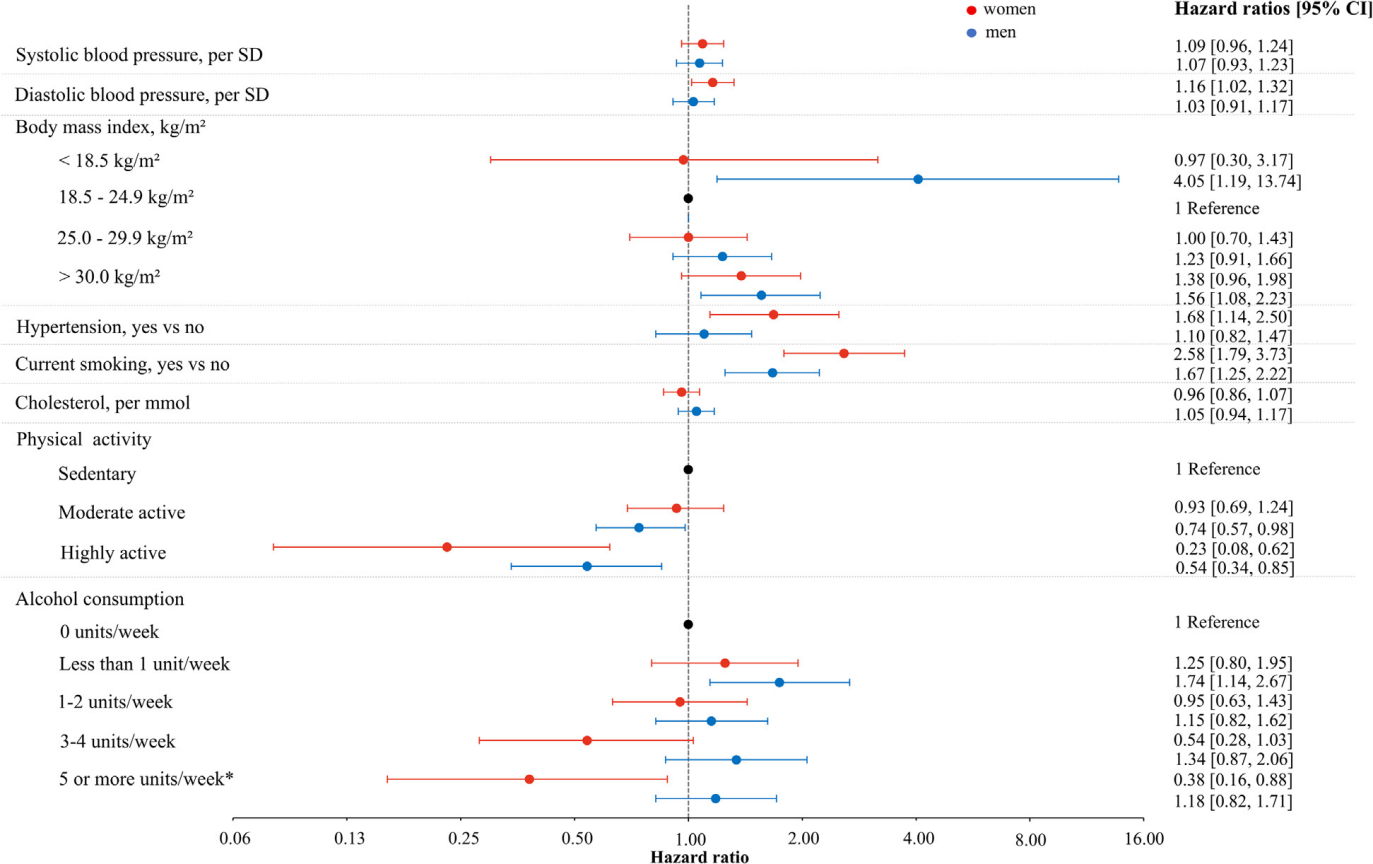
**HRs of Heart Failure in Women and Men With Atrial Fibrillation: The Tromsø Study, 1994 to 2016** Hypertension was defined as systolic blood pressure ≥140 mm Hg or diastolic blood pressure ≥90 mm Hg or current use of antihypertensive medications. HRs are adjusted for systolic blood pressure, body mass index, serum total cholesterol, current smoking, physical activity, history of myocardial infarction, stroke, and diabetes mellitus, and alcohol consumption, as well as age by using age as the time scale in the Cox regression models. ∗*P* < 0.05 for the difference between sexes calculated by including cross-product terms in the models.

In analyses exploring risk factors collected prior to AF diagnosis, current smoking was the only risk factor associated with subsequent HF in AF participants in both sexes (Figure 1). In women with AF, higher diastolic BP and hypertension increased HF risk, while among men with AF, a BMI below 18.5 kg/m^2^ or above 30.0 kg/m^2^ was associated with an increased risk of HF. Alcohol consumption of ≥5 per week was associated with a reduced risk of HF in women with AF, while men with AF who consumed <1 u of alcohol per week had an increased risk of incident HF compared to those who were abstinent. Higher physical activity levels were associated with reduced risk of HF. In women, the risk was only decreased for the highest level, while it was decreased for both moderately and highly active men. Among potential predictors, only alcohol intake of ≥5 u per week showed a significant interaction with sex.

### Combined impact of AF and HF on mortality

Mortality rates were lowest for participants without AF and HF, and highest for participants with paroxysmal/persistent AF and subsequent HF (Table 3). Compared to participants without AF and HF, women with AF (without subsequent HF) had a higher all-cause mortality risk regardless of AF subtype, whereas only men with paroxysmal/persistent AF had an increased risk. However, for cardiovascular mortality, permanent AF was associated with the highest risk in both sexes (Supplemental Table 2).

**Table 3:** HRs for Mortality According to Atrial Fibrillation and Heart Failure Status by Sex: The Tromsø Study, 1994 to 2016

	Women (n = 14,790)		*P* Value	Men (n = 13,181)			*P* Value^b^
	MR (95% CI)^a^	HR (95% CI)		MR (95% CI)^a^	HR (95% CI)	*P* Value	
Participants without AF/HF	3.2 (3.0-3.5)	1.00 (Reference)		4.7 (4.4-5.0)	1.00 (Reference)		
Paroxysmal/persistent AF	10.3 (8.7-12.2)	2.10 (1.78-2.48)	<0.001	12.2 (10.2-14.5)	1.66 (1.40-1.96)	<0.001	0.078
Permanent AF	6.3 (5.1-7.8)	1.40 (1.14-1.72)	0.001	8.5 (7.0-10.5)	1.16 (0.94-1.42)	0.161	0.259
Paroxysmal/persistent AF + HF	24.6 (19.2-31.4)	2.95 (2.30-3.77)	<0.001	30.8 (24.6-38.6)	3.42 (2.73-4.28)	<0.001	0.158
Permanent AF + HF	21.3 (17.6-25.8)	3.99 (3.32-4.80)	<0.001	26.6 (21.7-32.7)	2.88 (2.33-3.56)	<0.001	0.108

MR = mortality rate; other abbreviations as in Tables 1 and 2.

HRs are adjusted for systolic blood pressure, body mass index, serum total cholesterol, current smoking, alcohol consumption, physical activity, and history of myocardial infarction, stroke, and diabetes mellitus, as well as age by using age as the time scale in the Cox regression models.

a MR per 1,000 person-years, adjusted for a mean age of 45 y and calculating 95% CIs by using Poisson regression.

b *P* value for the difference between sexes calculated by including cross-product terms in the models.

For both AF subtypes and sexes, the risk of all-cause mortality increased further when HF developed (*P* < 0.001) (Table 3). In women, permanent AF before HF was associated with a higher risk of mortality than paroxysmal/persistent AF before HF. In men, however, paroxysmal/persistent AF prior to HF diagnosis was associated with the highest risk of all-cause mortality. The association between AF subtype with or without subsequent HF and all-cause mortality over the observational period is presented in Supplemental Figure 3. The trend observed for all-cause mortality was also evident for cardiovascular mortality (Supplemental Table 2).

## Discussion

In this large population-based cohort study, all AF subtypes were associated with an increased risk of developing HF, and the risk was highest for women with permanent AF. We found that current smoking was a common risk factor for incident HF in both sexes with AF. Increasing diastolic BP and hypertension were predictors for HF exclusively in women while being underweight or obese was associated with an increased risk of HF in men only. Participants who developed paroxysmal/persistent AF had the highest risk of death in both sexes when AF was the sole condition. Development of subsequent HF further enhanced the mortality risk (Central Illustration).

**Central Illustration:**
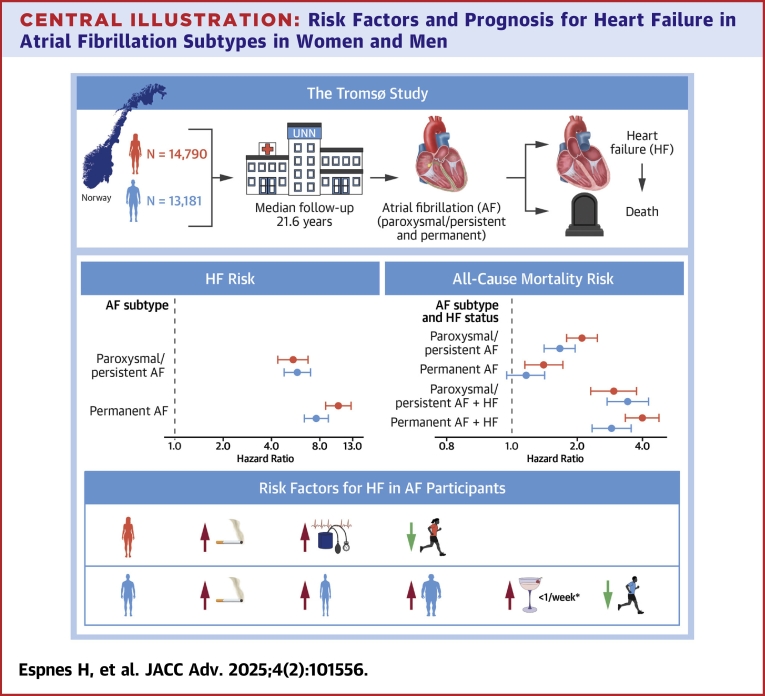
**Risk Factors and Prognosis for Heart Failure in Atrial Fibrillation Subtypes in Women and Men** HRs are adjusted for systolic blood pressure, body mass index, serum total cholesterol, smoking status, physical activity level, alcohol consumption, history of myocardial infarction, stroke, and diabetes mellitus, as well as age by using age as the time scale in the Cox regression models. Participants without atrial fibrillation and heart failure are used as the reference category. ∗Alcohol consumption of less than one unit per week was associated with increased risk compared to abstinence.

### Incident HF in participants with AF

AF is an established independent predictor of HF,^21^ and several studies indicate that permanent AF is associated with the highest risk of HF.^14^^,^^15^^,^^22^ In ORBIT-AF (Outcomes Registry for Informed Treatment of Atrial Fibrillation), permanent AF was associated with a 1.60 times higher risk for incident HF compared to paroxysmal AF.^14^ Additionally, permanent AF was associated with incident HF during 1-year follow-up in a study by Schnabel et al.^22^ Mogensen et al^23^ found that patients with paroxysmal AF had greater HF hospitalization risk compared to persistent/permanent AF. Importantly, outcomes were related to AF subtype in patients with established HF, so the results likely reflect the impact of AF on the course of HF. Our findings add to previous literature by showing that the risk of incident HF was substantially higher in those with permanent AF compared to persistent/paroxysmal AF. Furthermore, women with permanent AF had a higher relative risk of developing incident HF than men with permanent AF.

AF and HF are closely interrelated and impact each other in a vicious pathophysiological cycle.^24^ Irregular heart rate, loss of atrial systolic function, and neurohormonal activation due to AF can result in unfavorable hemodynamic effects, leading to left ventricular dysfunction and reduced cardiac output.^25^ These mechanisms facilitating HF in patients with AF are often more prominent in those with a higher AF burden and could explain why permanent AF was associated with a higher risk of HF in both sexes. In addition, sex differences in epidemiology and pathophysiology of both AF and HF have been previously described.^26^^,^^27^ Generally, women are diagnosed with these conditions later in life and are more likely to develop HF with preserved ejection fraction,^28^ while men are more prone to develop a combination of AF and HF at an earlier age, often accompanied by impaired left ventricular function secondary to previous myocardial infarction.^12^ There have also been noted differences in treatment approaches between sexes following an AF diagnosis. Previous literature has demonstrated that women are treated more conservatively regarding rhythm control and are less likely to receive electrical cardioversion and catheter ablation.^29^, ^30^, ^31^ Additionally, Gruber et al demonstrated that patients with AF undergoing catheter ablation have a lower risk of developing HF compared to those treated with anti-arrhythmic drugs.^32^ These disparities may influence the prognosis of AF subtypes and could explain some of the observed sex differences in HF risk.

### Modifiable risk factors for HF in participants with AF

Previous studies have demonstrated that well-established risk factors for HF development are also predictors of incident HF in patients with AF.^15^^,^^33^ Both hypertension and increased systolic and diastolic BP were risk factors for HF in participants without AF in our study (Supplemental Table 3). However, in participants with AF, hypertension and higher diastolic BP were associated with HF risk in women only. Pandey et al found an increased risk of HF in patients with AF with diastolic BP above 80 mm Hg.^14^ However, the association between systolic BP and increased risk of HF described previously^33^^,^^34^ was not found in our study. The relationship between BP and the subsequent risk of HF could have been affected by the initiation or intensification of antihypertensive treatment following the AF diagnosis. Women have been found to have a higher prevalence of hypertensive left ventricular hypertrophy which is less modifiable with BP-lowering medications than in males.^35^^,^^36^ These factors may contribute to the observed sex differences in the association between BP and risk of HF in our study.

Like others,^15^^,^^33^ we found that BMI was an independent predictor of HF in participants with AF. We also showed that BMI had a U-shaped relationship with the risk of HF in men, while only obesity was associated with increased risk of incident HF in women. Physical activity has been shown to reduce the risk of HF development.^37^ Contrary to the Women’s Health Study where no association between physical activity and risk of HF was identified,^34^ we found that highly active women with AF had reduced risk of subsequent HF. Additionally, both moderate and high activity were associated with decreased risk of HF in men.^38^^,^^39^

Previous studies suggest potential cardiovascular benefits from moderate alcohol consumption, including a reduced risk of incident HF.^38^^,^^39^ In our study, men consuming <1 u of alcohol per week had higher risk of HF than abstainers, while women consuming ≥5 u per week prior to AF diagnosis had a decreased risk of developing HF. The relationship between alcohol consumption and HF is complex. Our questionnaire on alcohol consumption may not capture specific details such as drinking patterns, types of beverages consumed, and exposure to alcohol over time. Additionally, our analysis may be limited by the modest drinking levels in Tromsø, reducing the statistical power to assess the impact of high alcohol consumption on HF risk.

### Risk of all-cause mortality amongst AF participants with and without HF

Although there have been conflicting results in the literature on the combined influence of AF and HF on adverse outcomes, recent research shows that the risk of mortality is increased when the 2 conditions coexist.^7^^,^^40^ In the Framingham Heart Study, the risk of mortality after AF increased by 2.7 in men and 3.1 in women when incident HF occurred.^7^ Ambriosio et al^41^ also found that the incidence of all-cause mortality was elevated in patients with AF and HF compared to those without HF. Supporting this, we demonstrated that patients with either AF subtype had a higher risk of mortality if they developed subsequent HF. Despite previous studies indicating worse outcomes for women with AF and HF,^7^^,^^12^ we found no sex differences in relative risk of mortality, regardless of AF subtype.

Previous research has shown that the risk of all-cause mortality was higher in nonparoxysmal AF than in paroxysmal AF.^42^^,^^43^ In contrast, except for in women with AF and subsequent HF, we found that participants with paroxysmal/persistent AF had a higher mortality risk than those with permanent AF. This could be related to the sudden changes in ventricular rate. Transient forms of AF might act as an indicator of HF instability, especially in patients with HF that can rapidly decompensate in response to sudden hemodynamic changes.^44^

### Strengths and limitations

Strengths of this study are its population-based design, access to repeated data on an individual level, standardized diagnostic criteria and validation of AF cases, high attendance over the surveys, and comprehensive collection of variables, allowing adjustment for multiple potential confounders. Additionally, the information on risk factors and confounders is gathered before the AF diagnosis, allowing to assess the risk factors before the potential influence of AF treatment.

Some limitations require acknowledgment. Several variables are self-reported which could lead to nondifferential misclassification. Although the AF diagnosis was validated, the AF pattern may have changed during follow-up. This would however only dilute the results. Contrary to AF diagnosis, an independent validation of all incident HF cases was not performed. However, 77 randomly selected participants with HF had their diagnosis validated, showing a positive predictive value of 88%,^45^ which is comparable to validation findings from other administrative databases.^46^ False positive HF cases could potentially lead to underestimation of the associations.

While acknowledging the potential issue of the multiple comparison problem in our results, we have presented the results as initially conducted. Many *P* values were <0.001, suggesting robust findings likely remain significant even with stricter significance thresholds. To address the risk of false positives in the risk factor analyses, we provided q-values (False Discovery Rate adjusted *P* values) for each risk factor (Supplemental Table 4). Despite some results exceeding a 5% false positive rate, there is also a risk of underestimating effects due to the reduced statistical power when conducting sex-specific analyses and categorizing variables. The same applies for the supplementary analyses accounting for competing risk of death. While the HF risk according to AF subtype (Supplemental Table 1) showed little change, several risk factors became nonsignificant (Supplemental Table 5), with larger *P* values and wider CIs, further suggesting power limitations. The results should therefore be interpreted with these considerations in mind.

Echocardiography data are not systematically available in the Tromsø Study for all HF cases to determine HF subtype. Furthermore, we do not have detailed information on medication use.

The generalizability of our findings may be limited due to participant recruitment from a single municipality in northern Norway. Furthermore, the 66% to 79% attendance introduces a potential response bias, as nonattendees have been shown to be slightly younger, have lower education and income levels, and more likely to be male and/or single compared to attendees.^16^^,^^47^

We do not have information from primary care, meaning patients with AF treated by their general practitioner only, are not included as an AF case in our data. In addition, due to their transient presentation possible cases with paroxysmal or persistent AF may be missed which could lead to an underestimation of the true population prevalence of AF.

## Conclusions

Our study provides new insights into the sex-specific relationship between AF subtypes and incident HF from a large, prospective community-based cohort. Regardless of sex, both AF subtypes were associated with an increased risk of HF, and for permanent AF, women showed higher relative risk than men. Smoking was a shared risk factor for HF in both sexes, while increased diastolic BP and hypertension were risk factors in women only, whereas being underweight or obese, or having low alcohol consumption were only risk factors in men. Subsequent development of HF was associated with an increased mortality in both sexes.

Perspectives **COMPETENCY IN CLINICAL KNOWLEDGE:** All AF subtypes increase HF risk, with women having permanent AF experiencing the highest risk. Development of subsequent HF elevates mortality risk across both sexes and AF subtypes. Smoking was a shared risk factor, while elevated diastolic BP and hypertension were risk factors only in women, whereas low alcohol consumption and being underweight or obese were risk factors only in men.

**TRANSLATIONAL OUTLOOK:** Further research is needed to investigate potential sex differences in the impact of HF subtypes and patient medications on these associations.

## Funding support and author disclosures

This study has been supported by internal fundings from UiT The Arctic University of Norway. Dr Schnabel has received funding from the 10.13039/501100000781European Research Council (ERC) under the European Union’s Horizon 2020 research and innovation programme under the grant agreement No 648131, from the European Union’s Horizon 2020 research and innovation programme under the grant agreement No 847770 (AFFECT-EU), from the European Union’s Horizon Europe research and innovation programme under the grant agreement ID: 101095480 and German Center for Cardiovascular Research (DZHK e.V.) (81Z1710103 and 81Z0710114); German Ministry of Research and Education (BMBF 01ZX1408A) and ERACoSysMed3 (031L0239). Wolfgang Seefried project funding 10.13039/501100005971German Heart Foundation. Dr Schnabel has received lectures fees and advisory board fees from BMS/Pfizer and Bayer outside this work. All other authors have reported that they have no relationships relevant to the contents of this paper to disclose.
