# Supplementary material for: Heart Failure in Atrial Fibrillation Subtypes in Women and Men in the Tromsø Study
Source: JACC Adv. 2025 Jan 10;4(2):101556. doi: 10.1016/j.jacadv.2024.101556 (PMC11773009; doi:10.1016/j.jacadv.2024.101556)
Supplement: Supplemental data [file mmc1.pdf]

SUPPLEMENTAL APPENDIX

Figure 1. Flow chart of study participants of the fourth, fifth and sixth survey. The Tromsø Study 1994-2016. 2

Figure 2. Cumulative incidence of heart failure according to subtype of atrial fibrillation by sex. The Tromsø Study 1994-2016. 3

Figure 3. Cumulative probability of all-cause mortality according to status of atrial fibrillation with and without heart failure by sex. The Tromsø Study 1994-2016. 4

Table 1. Subdistributed hazard ratios of incident heart failure according to atrial fibrillation subtype by sex. The Tromsø Study 1994-2016. 5

Table 2. Hazard ratios for cardiovascular mortality according to atrial fibrillation and heart failure status by sex. The Tromsø Study 1994-2016. 6

Table 3. Hazard ratios of heart failure in participants without atrial fibrillation by sex. The Tromsø Study 1994-2016. 7

Table 4. Hazard ratios of heart failure in participants with atrial fibrillation by sex with q-values. The Tromsø Study 1994-2016. 9

Table 5. Subdistributed hazard ratios of heart failure in participants with atrial fibrillation by sex. The Tromsø Study 1994-2016. 11

Figure 1. Flow chart of study participants of the fourth, fifth and sixth survey. The Tromsø Study 1994-2016.

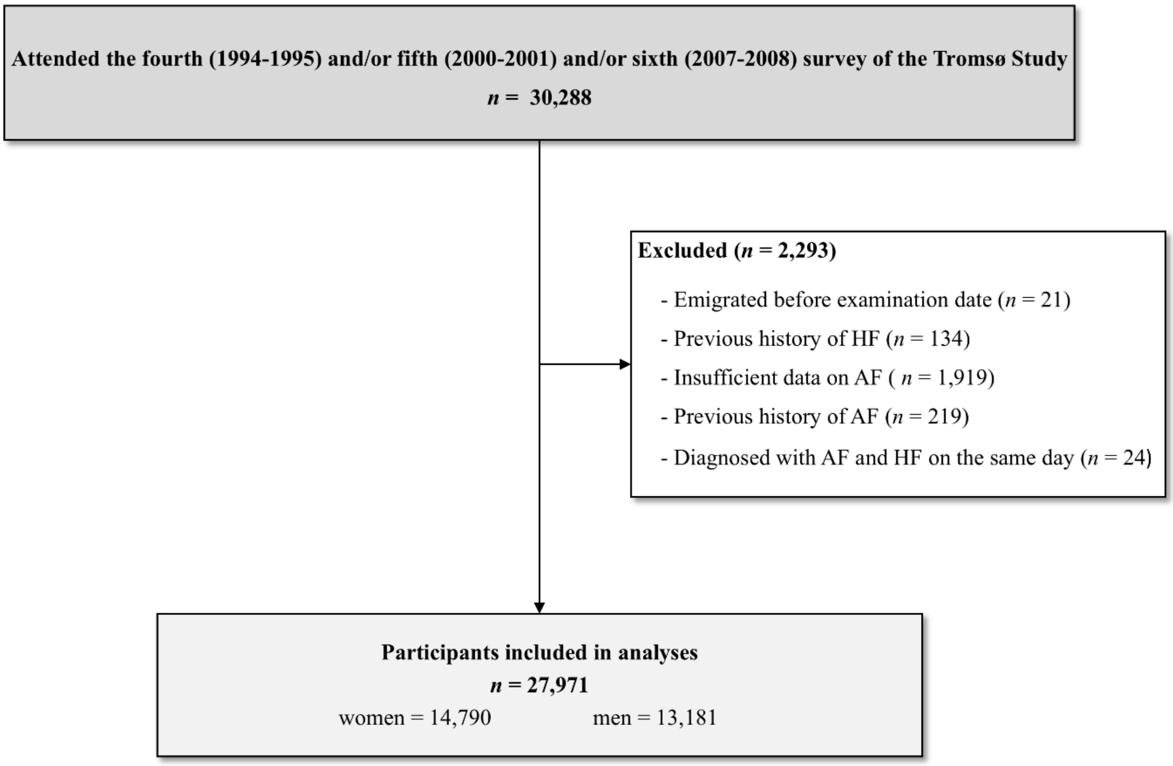

Figure 2. Cumulative incidence of heart failure according to subtype of atrial fibrillation by sex. The Tromsø Study 1994-2016.

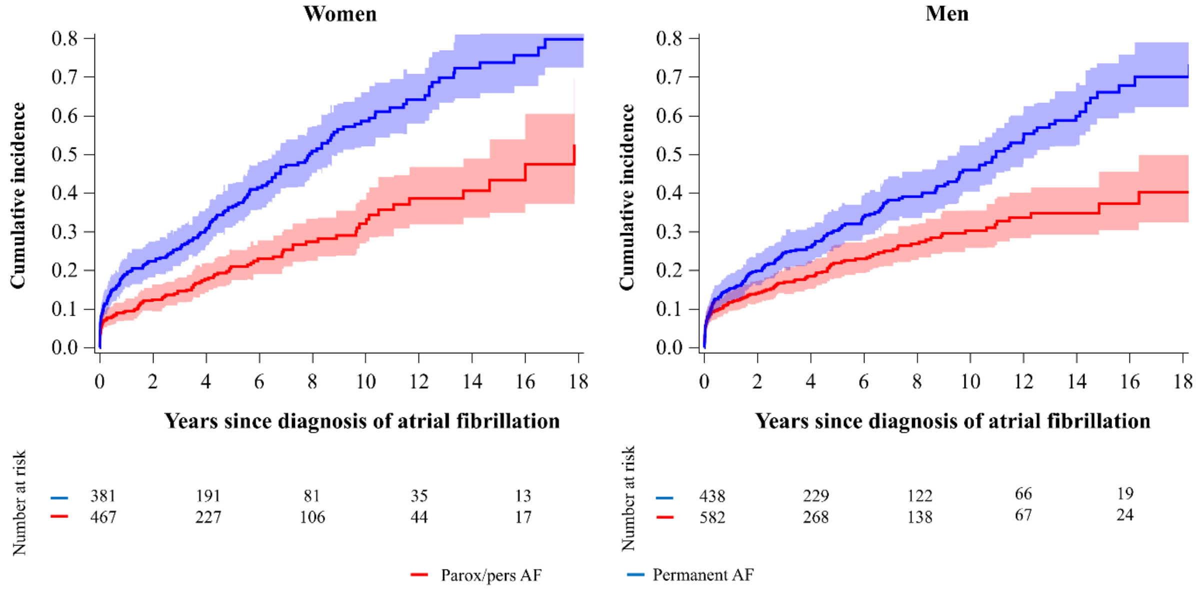

Models are adjusted for age, systolic blood pressure, body mass index, serum total cholesterol, smoking status, physical activity level, alcohol consumption, history of myocardial infarction, stroke, and diabetes mellitus.

AF indicates atrial fibrillation; parox, paroxysmal; pers, persistent.

Figure 3. Cumulative probability of all-cause mortality according to status of atrial fibrillation with and without heart failure by sex. The Tromsø Study 1994-2016.

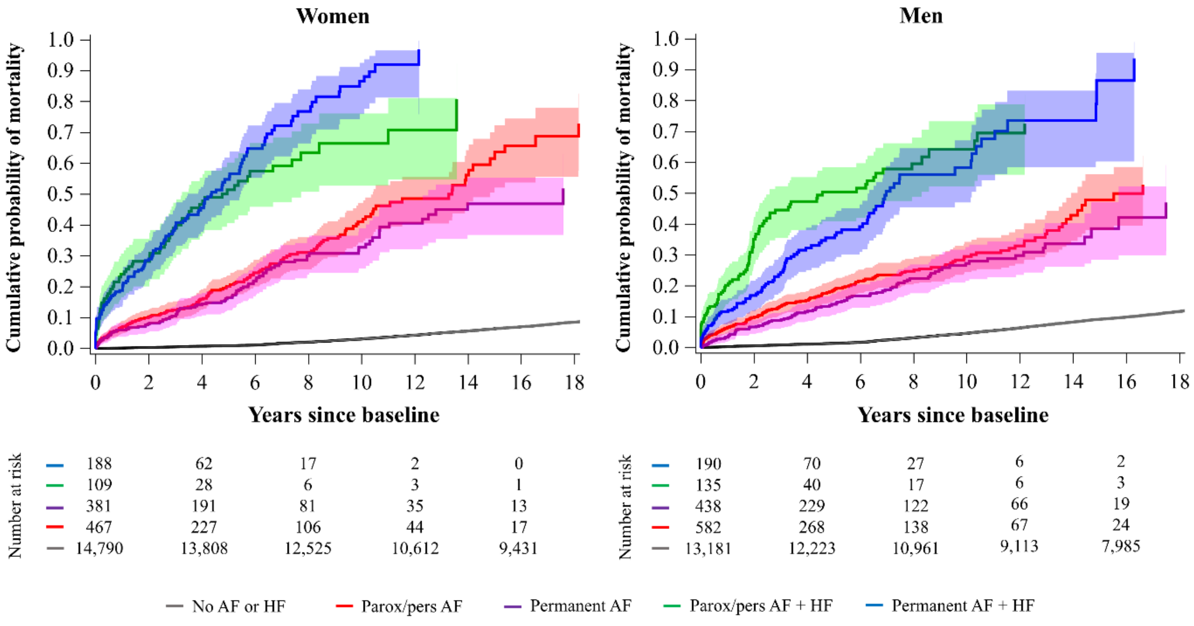

Models are adjusted for age, systolic blood pressure, body mass index, serum total cholesterol, smoking status, physical activity level, alcohol consumption, history of myocardial infarction, stroke, and diabetes mellitus. Participants without AF and HF are used as the reference category.

AF indicates atrial fibrillation; HF, heart failure; parox, paroxysmal; perm, permanent; pers, persistent.

Table 1. Subdistributed hazard ratios of incident heart failure according to atrial fibrillation subtype by sex. The Tromsø Study 1994-2016.

	Women *n* =14,790			Men *n* = 13,181			
	IR (95%CI)[a]	SHR (95% CI)	p-value	IR (95%CI)[a]	SHR (95% CI)	p-value	p-value[b]
Participants without AF	0.9 (0.8-1.0)	1 (Reference)		1.9 (1.7-2.2)	1 (Reference)		
Paroxysmal/persistent AF	6.4 (5.0-8.0)	4.38 (3.40-5.65)	<0.001	12.8 (10.5-15.6)	5.23 (4.14-6.61)	<0.001	0.604
Permanent AF	9.4 (7.6-11.6)	9.63 (7.73-11.99)	<0.001	16.7 (14.0-20.0)	8.03 (6.50-9.92)	<0.001	0.005

SHRs are obtained using the Fine-Gray subdistributed hazard model, and adjusted for systolic blood pressure, body mass index, serum total cholesterol, current smoking, alcohol consumption, physical activity, and history of myocardial infarction, stroke, and diabetes mellitus, as well as age by using age as the time scale in the regression models.
AF indicates atrial fibrillation; SHR, subdistributed hazard ratio; IR, incidence rate; CI, confidence interval.
[a]IR per 1,000 person-years, adjusted for a mean age of 45 years by using Poisson regression.
[b]p-value for the difference between sexes calculated by including cross-product terms in the models.

Table 2. Hazard ratios for cardiovascular mortality according to atrial fibrillation and heart failure status by sex. The Tromsø Study 1994-2016.

	**Women** *n* =14,790			**Men** *n* = 13,181			
	**MR (95% CI)[a]**	**HR (95% CI)**	**p-value**	**MR (95% CI)[a]**	**HR (95% CI)**	**p-value**	**p-value[b]**
Participants without AF/HF	1.0 (0.8-1.1)	1 (Reference)		1.7 (1.5-1.9)	1 (Reference)		
Paroxysmal/persistent AF	4.1 (3.3-5.2)	2.65 (2.16-3.24)	<0.001	6.4 (5.1-8.0)	2.23 (1.79-2.77)	<0.001	0.168
Permanent AF	4.7 (3.8-5.8)	3.21 (2.65-3.88)	<0.001	7.5 (6.0-9.3)	2.53 (2.05-3.12)	<0.001	0.056
Paroxysmal/persistent AF + HF	7.0 (4.9-9.9)	2.42 (1.71-3.41)	<0.001	12.5 (9.1-17.2)	3.53 (2.59-4.81)	<0.001	0.150
Permanent AF + HF	7.5 (5.8-9.7)	4.41 (3.48-5.59)	<0.001	13.9 (10.7-18.2)	3.33 (2.52-4.39)	<0.001	0.085

HRs are adjusted for systolic blood pressure, body mass index, serum total cholesterol, current smoking, alcohol consumption, physical activity, and history of myocardial infarction, stroke, and diabetes mellitus, as well as age by using age as the time scale in the Cox regression models.
AF indicates atrial fibrillation; CI, confidence interval; HF, heart failure; HR, hazard ratio; MR, mortality rate.
[a]MR per 1,000 person-years, adjusted for a mean age of 45 years and calculating 95% CIs by using Poisson regression.
[b]p-value for the difference between sexes calculated by including cross-product terms in the models.

Table 3. Hazard ratios of heart failure in participants without atrial fibrillation by sex. The Tromsø Study 1994-2016.

	Women *n* =14,709		Men *n* =13,181		
**Variable**	**HR (95% CI)**	**p-value**	**HR (95% CI)**	**p-value**	**p-value[a]**
Systolic blood pressure, per SD	1.15 (1.07-1.25)	<0.001	1.15 (1.05-1.25)	0.002	0.492
Diastolic blood pressure, per SD	1.12 (1.03-1.22)	0.006	1.15 (1.06-1.25)	0.001	0.805
Body mass index, kg/m^2					
< 18.5 kg/m^2	0.94 (0.42-2.14)	0.884	0.79 (0.20-3.19)	0.741	0.704
18.5 – 24.9 kg/m^2	1 (Reference)		1 (Reference)		
25.0 – 29.9 kg/m^2	1.16 (0.93-1.46)	0.185	1.14 (0.95-1.38)	0.163	0.832
> 30.0 kg/m^2	1.47 (1.13-1.92)	0.004	1.59 (1.23-2.07)	<0.001	0.559
Hypertension, yes vs no	1.35 (1.07-1.70)	0.010	1.42 (1.18-1.70)	<0.001	0.796
Current smoking, yes vs no	2.14 (1.73-2.65)	<0.001	1.81 (1.52-2.16)	<0.001	0.556
Cholesterol, per mmol/L	0.99 (0.92-1.07)	0.742	1.17 (1.10-1.26)	<0.001	0.008
Physical activity					
Sedentary	1 (Reference)		1 (Reference)		
Moderate active	0.86 (0.70-1.05)	0.132	0.71 (0.60-0.85)	<0.001	0.227
Highly active	0.76 (0.41-1.40)	0.374	1.08 (0.83-1.41)	0.557	0.234
Alcohol consumption, units/week					
0 units/week	1 (Reference)		1 (Reference)		
Less than 1 unit/week	0.82 (0.55-1.21)	0.310	0.81 (0.58-1.14)	0.217	0.975
1-2 units/week	0.70 (0.53-0.92)	0.011	0.77 (0.62-0.96)	0.018	0.445
3-4 units/week	0.74 (0.48-1.14)	0.167	0.72 (0.56-0.94)	0.016	0.969
5 or more units/week	0.44 (0.21-0.89)	0.022	0.80 (0.62-1.03)	0.080	0.086

Hypertension was defined as systolic blood pressure ≥140 mmHg or diastolic blood pressure ≥90 mmHg or current use of antihypertensive medications. HRs are adjusted for systolic blood pressure, body mass index, total cholesterol, current smoking, physical activity, history of myocardial infarction, stroke, and diabetes mellitus, and alcohol consumption, as well as age by using age as the time scale in the Cox regression models. When investigating systolic blood pressure, diastolic blood pressure and hypertension as risk factors, only one of these measurements was included in the models at a time. Participants with incident AF are included in the analyses until they were censored at the date of their AF diagnosis. [a]p-value for difference between sexes calculated by

including cross-product term in the models.
AF indicates atrial fibrillation; CI, confidence interval; HF, heart failure; HR, hazard ratio; n, numbers of observations; SD, standard deviation.

Table 4. Hazard ratios of heart failure in participants with atrial fibrillation by sex with q-values. The Tromsø Study 1994-2016.

	Women *n* =848			Men *n* =1,020		
**Variable**	**HR (95% CI)**	**p-value**	**q**	**HR (95% CI)**	**p-value**	**q**
Systolic blood pressure, per SD	1.09 (0.96-1.24)	0.167	0.292	1.07 (0.93-1.23)	0.327	0.470
Diastolic blood pressure, per SD	1.16 (1.02-1.32)	0.020	0.067	1.03 (0.91-1.17)	0.616	0.616
Body mass index, kg/m^2						
< 18.5 kg/m^2	0.97 (0.30-3.17)	0.964	0.999	4.05 (1.19-13.74)	0.025	0.07
18.5 – 24.9 kg/m^2	1 (Reference)			1 (Reference)		
25.0 – 29.9 kg/m^2	1.00 (0.70-1.43)	0.999	0.999	1.23 (0.91-1.66)	0.189	0.331
> 30.0 kg/m^2	1.38 (0.96-1.98)	0.087	0.174	1.56 (1.08-2.23)	0.017	0.060
Hypertension, yes vs no	1.68 (1.14-2.50)	0.009	0.042	1.10 (0.82-1.47)	0.533	0.574
Current smoking, yes vs no	2.58 (1.79-3.73)	<0.001	0.001	1.67 (1.25-2.22)	<0.001	0.001
Cholesterol, per mmol/L	0.96 (0.86-1.07)	0.455	0.637	1.05 (0.94-1.17)	0.357	0.470
Physical activity						
Sedentary	1 (Reference)			1 (Reference)		
Moderate/active	0.93 (0.69-1.24)	0.618	0.787	0.74 (0.57-0.98)	0.033	0.077
Highly active	0.23 (0.08-0.62)	0.004	0.028	0.54 (0.34-0.85)	0.008	0.047
Alcohol consumption, units/week						
0 units/week	1 (Reference)			1 (Reference)		
Less than 1 unit/week	1.25 (0.80-1.95)	0.339	0.527	1.74 (1.14-2.67)	0.010	0.047
1-2 units/week	0.95 (0.63-1.43)	0.813	0.949	1.15 (0.82-1.62)	0.424	0.495
3-4 units/week	0.54 (0.28-1.03)	0.060	0.140	1.34 (0.87-2.06)	0.183	0.331
5 or more units/week	0.38 (0.16-0.88)	0.024	0.067	1.18 (0.82-1.71)	0.369	0.470

AF indicates atrial fibrillation; HF, heart failure; HR, hazard ratio; CI, confidence interval; SD, standard deviation; q, q-value. Hypertension was defined as systolic blood pressure ≥140 mmHg and/or diastolic blood pressure ≥90 mmHg and/or current use of antihypertensive medications. HRs are adjusted for systolic blood pressure, body mass index, serum total cholesterol, current smoking, physical activity, history of myocardial infarction, stroke, and diabetes mellitus, and alcohol consumption, as well as age by using age as the time scale in the Cox regression models. When investigating systolic blood pressure, diastolic blood pressure and hypertension as risk factors, only one of these

measurements was included in the models at a time. Q-values are calculated using the Benjamini-Hochberg procedure for adjusting p-values.

Table 5. Subdistributed hazard ratios of heart failure in participants with atrial fibrillation by sex. The Tromsø Study 1994-2016.

	Women *n* =14,709		Men *n* =13,181		
**Variable**	**SHR (95% CI)**	**p-value**	**SHR (95% CI)**	**p-value**	**p-value[a]**
Systolic blood pressure, per SD	1.09 (0.95-1.25)	0.217	1.10 (0.94-1.29)	0.252	0.499
Diastolic blood pressure, per SD	1.14 (0.99-1.32)	0.073	1.03 (0.90-1.19)	0.649	0.307
Body mass index, kg/m^2					
< 18.5 kg/m^2	0.87 (0.25-3.01)	0.830	3.20 (0.84-12.22)	0.090	0.334
18.5 – 24.9 kg/m^2	1 (Reference)		1 (Reference)		
25.0 – 29.9 kg/m^2	1.17 (0.79-1.73)	0.447	1.13 (0.81-1.58)	0.482	0.204
> 30.0 kg/m^2	1.43 (0.95-2.15)	0.084	1.30 (0.87-1.93)	0.203	0.463
Hypertension, yes vs no	1.60 (1.04-2.48)	0.034	1.15 (0.83-1.58)	0.408	0.148
Current smoking, yes vs no	1.48 (0.98-2.24)	0.064	1.22 (0.89-1.67)	0.211	0.608
Cholesterol, per mmol/L	0.95 (0.85-1.06)	0.332	1.07 (0.95-1.20)	0.258	0.519
Physical activity					
Sedentary	1 (Reference)		1 (Reference)		
Moderate active	0.82 (0.59-1.14)	0.244	0.87 (0.64-1.18)	0.372	0.697
Highly active	0.27 (0.10-0.75)	0.012	0.65 (0.39-1.10)	0.111	0.143
Alcohol consumption, units/week					
0 units/week	1 (Reference)		1 (Reference)		
Less than 1 unit/week	1.20 (0.73-2.00)	0.474	1.68 (1.02-2.78)	0.042	0.487
1-2 units/week	0.85 (0.54-1.34)	0.495	1.16 (0.80-1.67)	0.446	0.760
3-4 units/week	0.70 (0.36-1.33)	0.275	1.09 (0.69-1.74)	0.712	0.028
5 or more units/week	0.32 (0.13-0.76)	0.012	1.05 (0.70-1.57)	0.823	0.013

Hypertension was defined as systolic blood pressure ≥140 mmHg or diastolic blood pressure ≥90 mmHg or current use of antihypertensive medications. SHRs are obtained using the Fine-Gray subdistributed hazard model, and adjusted for systolic blood pressure, body mass index, total cholesterol, current smoking, physical activity, history of myocardial infarction, stroke, and diabetes mellitus, and alcohol consumption, as well as age by using age as the time scale in the regression models. When investigating systolic blood pressure, diastolic blood pressure and hypertension as risk factors, only one of these measurements was included in the models at a

time. [a]p-value for difference between sexes calculated by including cross-product term in the models.
AF indicates atrial fibrillation; CI, confidence interval; HF, heart failure; SHR, subdistributed hazard ratio; n, numbers of observations; SD, standard deviation.
